# Supplementary material for: Modes of Transport to School and Their Associations with Weight Status: A Cross-Sectional Survey of Students in Shanghai, China
Source: Int J Environ Res Public Health. 2021 Apr 28;18(9):4687. doi: 10.3390/ijerph18094687 (PMC8124258; doi:10.3390/ijerph18094687)
Supplement: Supplementary file 1 [file ijerph-18-04687-s001.zip › ijerph-1141136-supplementary.pdf]

SUPPLEMENTAL FIGURES AND TABLES

Table S1 Demographic distribution of students with different travel modes.

Characteristics	By public transport or bike or walking (N= 4710) n (%)	Vehicle passengers (N= 1779) n (%)	Bike passengers (N= 1309) n (%)	Mixed mode (N= 2325) n (%)	*p* value *
**Gender**					<0.001
male	2482(52.7)	761(42.8)	666(50.9)	1086(46.7)	
female	2228(47.3)	1018(57.2)	643(49.1)	1239(53.3)	
**Age-groups**					<0.001
First to third grade	1372(29.1)	358(20.1)	522(39.9)	591(25.4)	
Fourth to fifth grade	693(14.7)	237(13.3)	284(21.7)	338(14.5)	
Sixth to ninth grade	1476(31.3)	630(35.4)	435(33.2)	790(34.0)	
Tenth to twelfth grade	1169(24.8)	554(31.1)	68(5.2)	606(26.1)	
**Living area**					<0.001
Core	2560(54.4)	390(21.9)	545(41.6)	777(33.4)	
Extended	2150(45.6)	1389(78.1)	764(58.4)	1548(66.6)	
**Family educational level**					<0.001
Up to secondary education (year 12)	1163(24.7)	301(16.9)	382(29.2)	482(20.7)	
Junior college degree	1148(22.4)	470(26.4)	343(26.2)	610(26.2)	
Bachelor or above	2399(50.9)	1008(56.7)	584(44.6)	1233(53.0)	
**Superior occupation social class**					<0.001
neither	2256(47.9)	732(41.1)	704(53.8)	1082(46.5)	
only in fathers	1084(23.0)	370(20.8)	279(21.3)	545(23.4)	
only in mothers	313(6.6)	172(9.7)	87(6.6)	154(6.6)	
both	1057(22.4)	505(28.4)	239(18.3)	544(23.4)	

* Pearson chi-square tests

Table S2 Relative odds of underweight, overweight, and obese in health behavioral factors.

Characteristics	Underweight OR (95%*CI*)	Overweight OR (95%*CI*)	Obese OR (95%*CI*)
**Gender**			
Male	1.00	1.00	1.00
Female	0.79(0.66, 0.94)	0.54(0.48, 0.60)	0.44(0.39, 0.50)
**Age-groups**			
First to third grade	1.00	1.00	1.00
Fourth to fifth grade	0.85(0.62, 1.17)	1.20(1.01, 1.42)	1.10(0.93, 1.30)
Sixth to ninth grade	1.15(0.91, 1.46)	1.31(1.14, 1.51)	0.86(0.75, 1.00)
Tenth to twelfth grade	1.27(1.00, 1.62)	0.99(0.85, 1.16)	0.55(0.47, 0.66)
**Living area**			
Core	1.00	1.00	1.00
Extended	0.87(0.73,1.04)	0.85(0.76,0.95)	0.82(0.73,0.92)
**Family educational level**			
Up to secondary education (year 12)	1.00	1.00	1.00
Junior college degree	1.01(0.78, 1.30)	0.99(0.85, 1.15)	0.93(0.79, 1.09)
Bachelor or above	0.93(0.74, 1.17)	0.83(0.73, 0.95)	0.89(0.77, 1.02)
**Superior occupation social class**			
Neither	1.00	1.00	1.00
Only in fathers	1.22(0.97, 1.52)	0.92(0.80, 1.05)	0.94(0.81, 1.08)
Only in mothers	1.34(0.95, 1.89)	1.10(0.89, 1.36)	1.11(0.88, 1.38)
Both	1.07(0.85, 1.35)	0.88(0.77, 1.01)	0.86(0.75, 1.00)
**Modes of transport to school**			
Active mode	1.00	1.00	1.00
Vehicle passengers	1.10(0.87, 1.40)	0.78(0.68, 0.91)	0.74(0.63, 0.88)
Bike passengers	1.10(0.84, 1.45)	0.98(0.83, 1.15)	1.00(0.84, 1.20)
Mixed mode	0.90(0.71, 1.14)	0.83(0.73, 0.96)	0.90(0.78, 1.04)

Figure S1 Distribution of sampling schools on the map. The light orange area is the urban area of Shanghai (within the outer ring road). Each point in the graph represents a school, and the points of different colors represent schools of different stages.

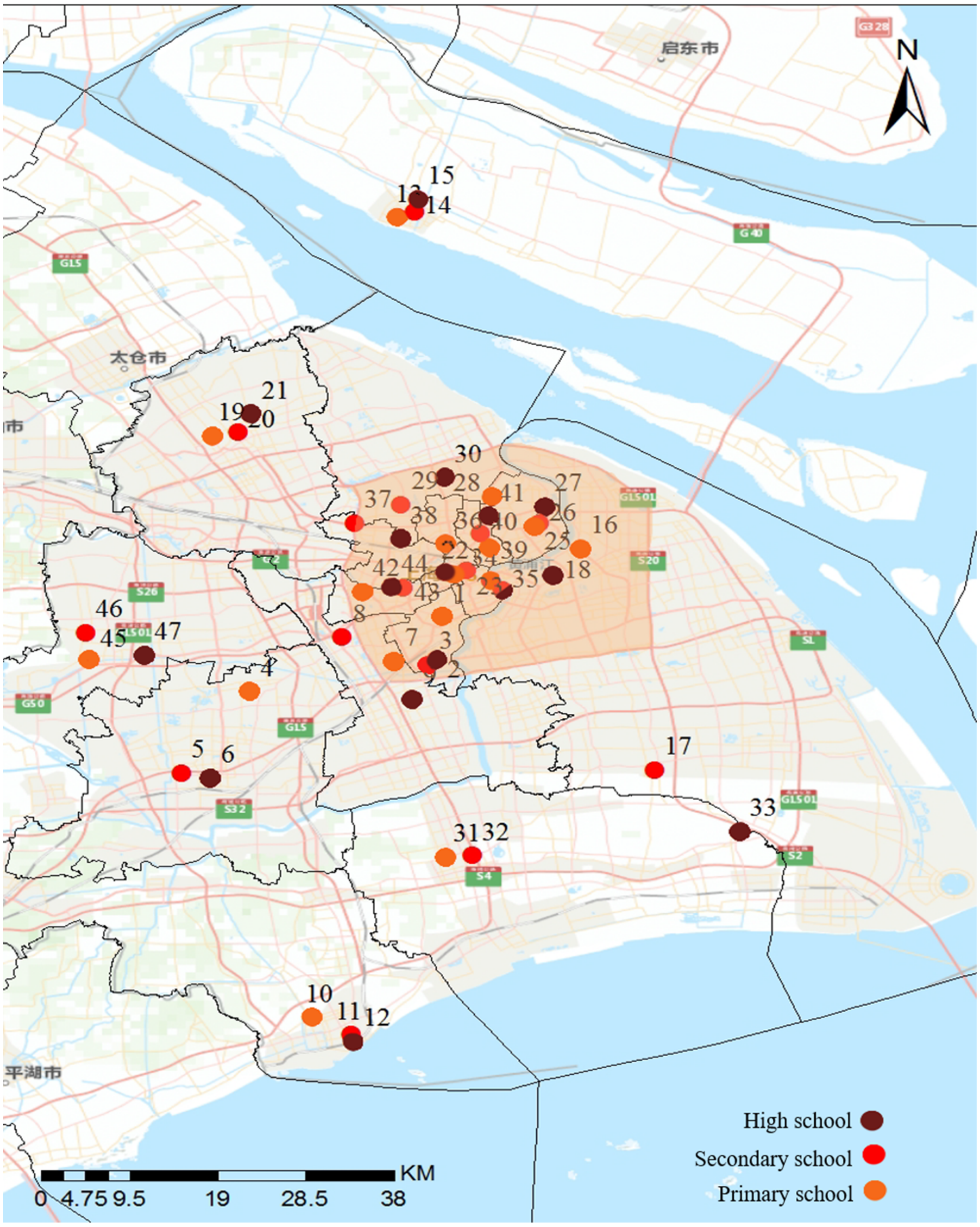

Figure S2 Directed acyclic graphs (DAGs) for the association between previous modes of transport to school with childhood overweight/obesity. Variables with superscript "a" mean exposure, that with superscript "b" means outcome, and those with superscript "c" mean ancestors of exposure. Variables with dashed rectangle are collisions.

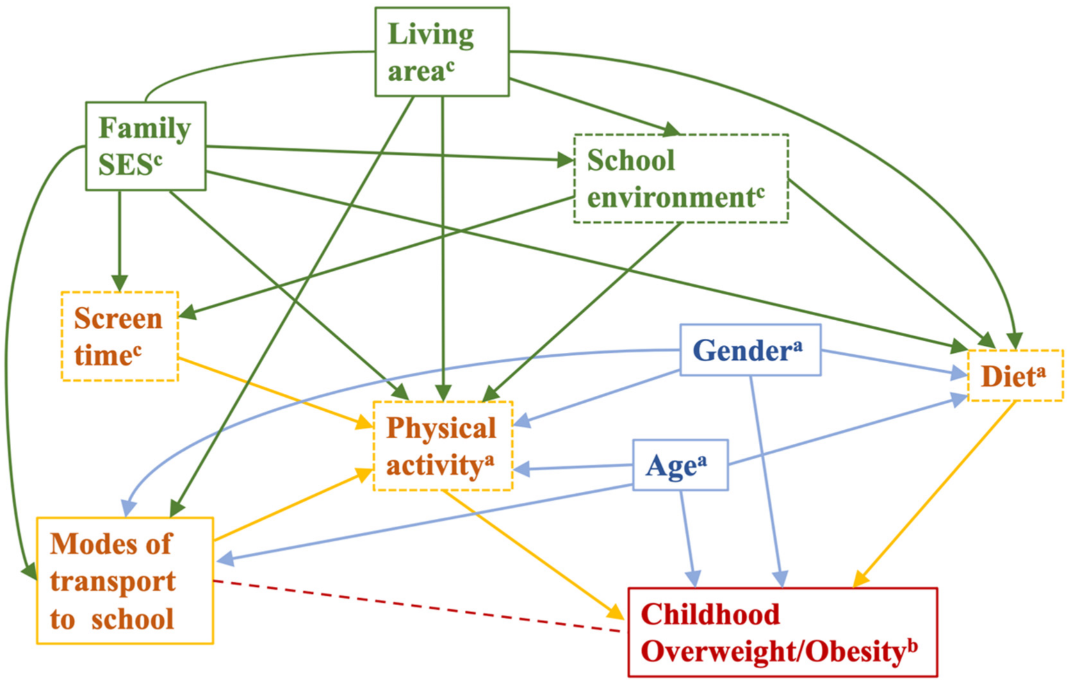

Figure S3 Travel mode share in different groups. (a) grouped by area (b) grouped by age

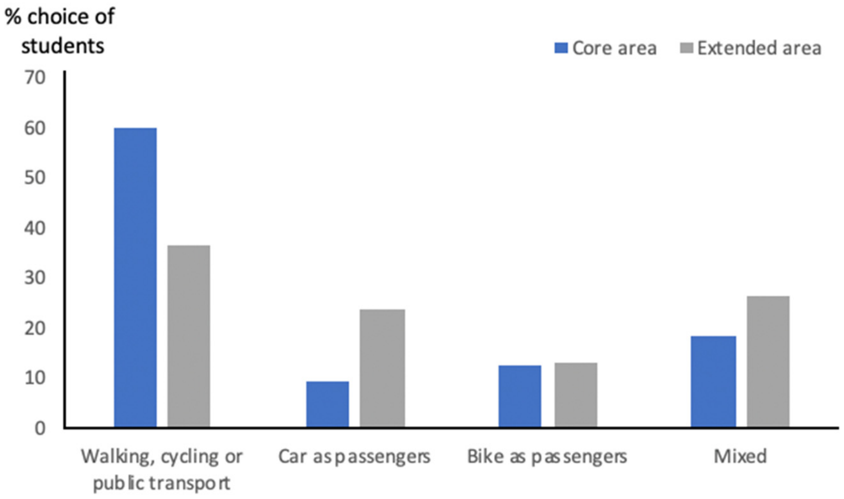

(a)

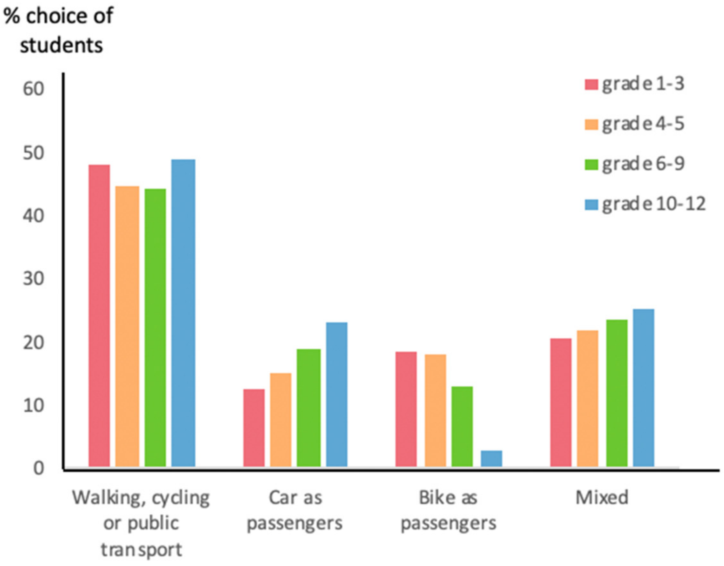

(b)
